# Investigating the functionality of a ribosome-binding mutant of NAA15 using *Saccharomyces cerevisiae*

**DOI:** 10.1186/s13104-018-3513-4

**Published:** 2018-06-22

**Authors:** Sylvia Varland, Thomas Arnesen

**Affiliations:** 1 Department of Biological Sciences, University of Bergen, 5006 Bergen, Norway; 2 Department of Biomedicine, University of Bergen, 5009 Bergen, Norway; 3 Donnelly Centre for Cellular and Biomolecular Research, University of Toronto, Toronto, ON M5S 3E1 Canada; 4 Department of Surgery, Haukeland University Hospital, 5021 Bergen, Norway

**Keywords:** N-terminal acetyltransferase, NatA, NAA10, NAA15, N-terminal acetylation, Ribosome association, *Saccharomyces cerevisiae*

## Abstract

**Objective:**

N-terminal acetylation is a common protein modification that occurs preferentially co-translationally as the substrate N-terminus is emerging from the ribosome. The major N-terminal acetyltransferase complex A (NatA) is estimated to N-terminally acetylate more than 40% of the human proteome. To form a functional NatA complex the catalytic subunit NAA10 must bind the auxiliary subunit NAA15, which properly folds NAA10 for correct substrate acetylation as well as anchors the entire complex to the ribosome. Mutations in these two genes are associated with various neurodevelopmental disorders in humans. The aim of this study was to investigate the in vivo functionality of a *Schizosaccharomyces pombe* NAA15 mutant that is known to prevent NatA from associating with ribosomes, but retains NatA-specific activity in vitro.

**Results:**

Here, we show that *Schizosaccharomyces pombe* NatA can functionally replace *Saccharomyces cerevisiae* NatA. We further demonstrate that the NatA ribosome-binding mutant Naa15 ΔN K6E is unable to rescue the temperature-sensitive growth phenotype of budding yeast lacking NatA. This finding indicates the in vivo importance of the co-translational nature of NatA-mediated N-terminal acetylation.

## Introduction

N-terminal acetylation is one of the most common protein modifications in eukaryotes, occurring on approximately 80–90% of all human proteins [1–3]. The functional consequences of attaching an acetyl group to the N-terminus of proteins are diverse. N-terminal acetylation plays a role in regulating protein properties such as folding [4–9], stability [10, 11], subcellular localization [12–14], complex formation [15, 16], complex stoichiometry [11] as well as regulating gene expression [17, 18], and actin cytoskeleton dynamics [19]. Moreover, N-terminal acetylation is crucial for normal development of multicellular organisms [20–23]. N-terminal acetylation is carried out by a family of enzymes called N-terminal acetyltransferases (NATs). The biological importance of this protein modification is underscored by the fact that dysfunctional NATs are implicated in a variety of developmental disorders and cancers [24–33].

In most cases the NATs anchor to the ribosome where they act on nascent polypeptides as they emerge from the exit tunnel during translation [1]. The enzyme complex NatA is one of the main contributors of N-terminal acetylation [34, 35]. It acetylates N-termini after the initiator methionine has been cleaved of by methionine aminopeptidase, thus exposing a small N-terminal amino acid (Ser, Ala, Gly, Thr, Val or Cys) [35–37]. The NatA complex is composed of the catalytic subunit Naa10 (Ard1 in yeast) and the auxiliary subunit Naa15 (Nat1) [36, 38–40], both of which are evolutionarily conserved [35]. Structure analysis of the NatA complex from the fission yeast *Schizosaccharomyces pombe* (Sp) revealed that Naa15 contains 13 conserved tetratricopeptide repeats (TPR) that wraps around Naa10 in a ring-like manner [41]. The binding of Naa15 induces an allosteric change in the active site of Naa10, which is essential for catalysis by the NatA complex. Thus, Naa15 is considered a regulatory switch that controls NatA activity. Moreover, Naa15 mediates ribosomal anchoring and interacts with nascent polypeptide [40]. It is thought that Naa15 binds to the general docking site for ribosome-associated factors Rpl25/35 (L23/L29), which is favorable positioned in close proximity to the ribosomal exit tunnel [42, 43].

To gain a better understanding for the molecular basis that underlies the interaction between NATs and the ribosome, Magin and colleagues carried out a conservation and electrostatic surface analysis of NatA [44]. Focusing on Naa15, they identified two conserved electropositive regions (EPR) on the surface of NatA that appeared responsible for ribosome interaction. Both regions are situated on the same side of the enzyme and would optimally position the active site of ribosomal-bound Naa10 to emerging nascent polypeptides. EPR1 is located within the N-terminal domain of Naa15 and includes the first three TPRs while EPR2 consist of an internal basic α-helix that is situated near the C-terminus. By performing mutation analyses of EPR1 and EPR2 the authors generated a SpNaa15 mutant that retained its ability to bind Naa10 and further full enzymatic activity in vitro, but was unable to associate with ribosomes [44]. The functional impact of this ribosome-binding mutant of SpNaa15 in vivo is yet to be investigated.

In this study, we have investigated the functionality of a SpNaa15 mutant that is unable to bind ribosomes. We show, using the budding yeast *Saccharomyces cerevisiae* (Sc) as a model, that EPR1 and EPR2 contain important functional regions required for NatA activity in vivo. This study highlights the importance of NatA-mediated N-terminal acetylation taking place during protein synthesis.

## Main text

### Methods

#### Yeast strains, plasmid construction, and transformation

The *Saccharomyces cerevisiae* strain W303-1A (*MAT*a; *ade2*-*1*; *ura3*-*1*; *his3*-*11*,*15*; *leu2*-*3*,*112*; *trp1*-*1*; *can1*-100) was used to construct a *ScNatA*Δ strain (*ard1*-Δ*::LEU2; nat1*-Δ*::kanMX*) [35]. Gene deletions were verified by colony PCR using primers ARD1-176 F (5′-GTCTTTATTGATCTCTAGGCTCAATCC-3′) with ARD1 848 R (5′-CCTTACTATTCATGCTCACACAATTC-3′) and NAT1 -213 F (5′-CCAAATTGCATGACCTTGCTAATGAGG-3′) with NAT1 2763 R (5′-GGAAAGCAAGAATTTTGGCAAGAAAAGG-3′). The *S. cerevisiae* expression vector pBEVY-U-SpNatA was generated by inserting a C-terminally truncated version of *SpNAA10*-V5 (residues 1–156 out of 177 total residues) after the ADH1 promoter using the *Xma*I/*Eco*RI sites and full-length HA-*SpNAA15* (residues 1–729) after the GPD promoter using the *Xba*I/*SalI* sites. pBEVY-U-SpNatA was used to construct pBEVY-U-SpNatA-ΔN-K6E (p.SpNaa15 aa1_109del, K605E, K606E, K609E, K610E, K612E, K613E) in a three-step process using the Q5 site-directed mutagenesis kit (NEB, #E0554S) with the following mutagenic primers: (i) SpNAA15 aa1_109 del F (5′-AACAACTCGAGTCTTTTGCG-3′) with SpNAA15 aa1_109 del R (5′-AAGGGCCTGTACAGCGTAAT-3′), (ii) SpNAA15 aa605_613del F (5′-GACCTTAGTAAACGATTGGAACG-3′) with SpNAA15 aa605_613del R (5′-TTCCTCTTCTTCATTTATTTCTCCAC-3′), and (iii) SpNAA15 aa605_613ins F (5′-agaactcgaagaaGACCTTAGTAAACGATTGG-3′) with SpNAA15 aa605_613ins R (5′-tcataaatttcttcTTCCTCTTCTTCATTTATTTCTC-3′). Mutants were confirmed by sequencing. pBEVY-U-SpNatA and pBEVY-U-SpNatA-ΔN-K6E were transformed into the *ScNatA*Δ strain using standard yeast techniques [45]. In addition, the wild-type strain and the *ScNatA*Δ strain were transformed with empty pBEVY-U plasmid. Transformants were selected and maintained on SD-Ura agar [0.67% (w/v) yeast nitrogen base without amino acids, 0.2% (w/v) yeast drop-out mix without uracil, 2% (w/v) glucose, and 2% (w/v) agar].

#### Immunoblotting

Yeast whole-cell protein extracts were prepared by alkaline treatment as previously described [46]. Protein extracts were separated by SDS-PAGE and analyzed by immunoblotting. The immunoblots were probed with rabbit polyclonal anti-HA tag (1:10,000, Abcam, ab9110), mouse monoclonal anti-V5 tag (1:10,000, Invitrogen, #R960-25), and rabbit polyclonal anti-Zwf1 (1:15,000, Sigma, A9521). HRP-conjugated rabbit or mouse anti-goat IgG were used as secondary antibodies (1:10,000, Bio-Rad, #1706515 and #1706516). All antibodies were diluted in 1X TBS containing 5% nonfat dry milk (w/v) and 0.05% Tween-20. The immunoblots were developed using SuperSignal West Femto Maximum Sensitivity Substrate (Thermo Scientific, #34095) followed by detection and imaging using VersaDoc MP 5000 from Bio-Rad.

#### Yeast growth assay

Wild-type and *ScNatA*Δ yeast strains were grown in SD-Ura at 30 °C to early log phase (OD_600_ 0.8–1.0) and adjusted to 1 OD_600_/ml. Ten-fold serial dilutions with sterile milliQ water were spotted (2 µl) onto YPD [1% (w/v) yeast extract, 2% (w/v) peptone (w/v), 0.012% (w/v) adenine, 2% (w/v) glucose, and 2% (w/v) agar] and SD-Ura agar [0.67% (w/v) yeast nitrogen base without amino acids, 0.2% (w/v) yeast drop-out mix without uracil, 2% (w/v) glucose, and 2% (w/v) agar]. The plates were incubated at 30 or 38 °C for 2 days and imaged with spImager from S&P Robotics.

### Results and discussion

**Fig. 1:**
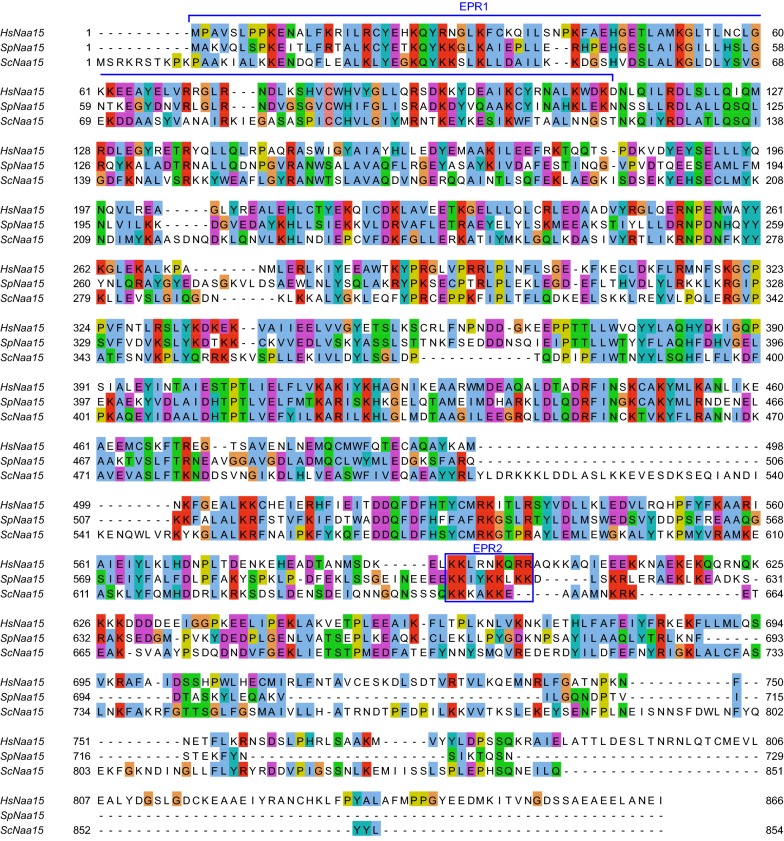
Multiple sequence alignment of Naa15 from *H. sapiens* (Hs), *S. pombe* (Sp), and *S. cerevisiae* (Sc). The alignment was generated in Clustal Omega [51] and edited in Jalview [52]. The N-terminal region (EPR1) and the internal basic helix (EPR2) are indicated in blue. Light blue color indicates hydrophobic residues, red indicates basic residues, magenta indicates acidic residues, green indicates polar residues, pink indicates cysteines, orange indicates glycines, yellow indicates prolines, and cyan indicates aromatic residues

**Fig. 2:**
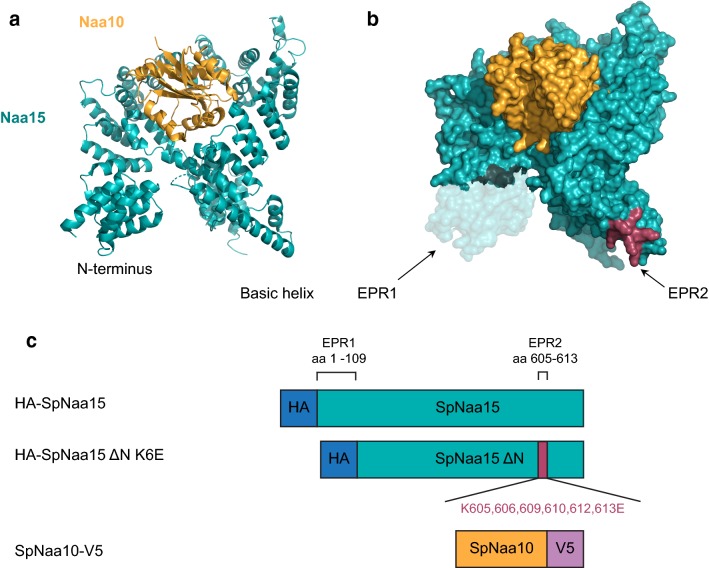
Schematic representation of the SpNatA structure and constructs used. **a** Cartoon and **b** surface representation of the SpNatA complex, showing SpNaa15 in teal and SpNaa10 in orange. The N-terminal region (transparent teal) and the internal basic helix (raspberry) are indicated. The SpNatA structure was generated from PDB ID: 4KVO using PyMOL version 2.0 Schrödinger, LLC. **c** Schematic of the SpNatA constructs used with the two predicted electropositive regions (EPR1 and 2) indicated. Blue, HA-tag; teal, SpNaa15; raspberry, mutated amino acid residues; orange, SpNaa10; purple, V5-tag. Note that for SpNaa10 only residues 1–156 out of the 177 total residues were used

Naa15 constitutes the auxiliary part of the NatA complex and regulates its activity in a dual manner: by anchoring the catalytic subunit Naa10 to the ribosome so that nascent polypeptide chains are presented to Naa10 which in turn acetylates the N-terminal amino group, and secondly by modulating the catalytic site of Naa10 to match the broad spectrum of its in vivo substrates [40]. Both Naa10 and Naa15 are highly conserved in eukaryotes [35]. The human Naa15 protein share 46.6% and 43.4% sequence similarity with its *S. pombe* and *S. cerevisiae* homologues, respectively, with largest variation at the C-terminus (Fig. 1). The sequence similarity between SpNaa15 and ScNaa15 is 44.5%. Using the structure of SpNatA (Fig. 2a) [41], Magin et al. [44] identified two conserved electropositive regions (EPR) in Naa15, an N-terminal region and an internal basic helix near the C-terminus, that could potentially facilitate the interaction between NatA and the ribosomes. Moreover, they generated a series of mutants targeting these two regions. They showed, using an in vitro N-terminal acetylation assay, that the NatA variant SpNaa15 ΔN K6E (p.Δ1-109, K605E, K606E, K609E, K610E, K612E, K613E) (Fig. 2b) was enzymatically active towards the serine-starting peptide SESS-(corresponding to the N-terminus of HMGA1), representing a classical NatA substrate. They also revealed with NatA–ribosome co-sedimentation and gel filtration analyses that the ΔN K6E mutant was unable to bind ribosomes.

**Fig. 3:**
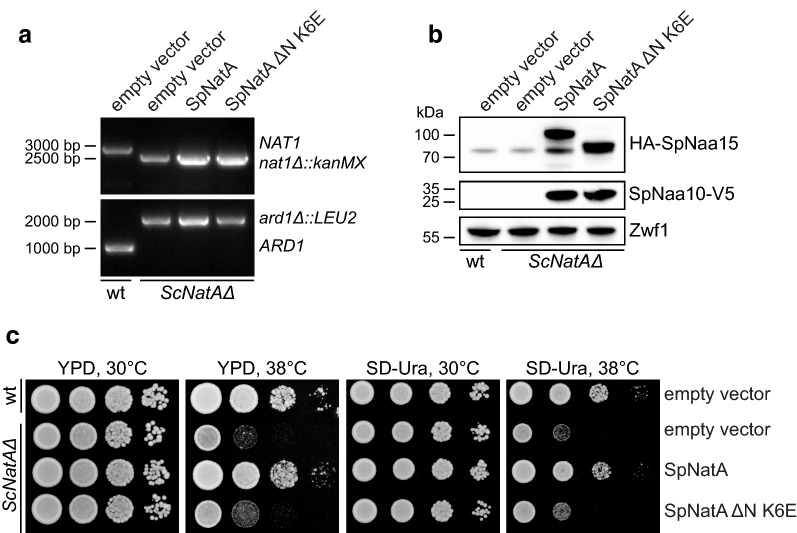
SpNatA ΔN-K6E does not rescue growth of *ScNatA*Δ cells at high temperature. **a** Confirming gene disruption of *ARD1* and *NAT1* in a *ScNatA*Δ strain by colony PCR using *ARD1* and *NAT1* primers. *ARD1*-specific PCR product 1024 bp (*ARD1* 717 bp + gene-specific sequence 307 bp). *NAT1*-specific PCR product 2976 bp (*NAT1* 2565 bp + gene-specific sequence 411 bp). **b** SpNatA expression was confirmed by immunoblot analyses using anti-HA (to detect HA-SpNaa15) and anti-V5 (to detect SpNaa10-V5). Anti-Zwf1 served as loading control. **c** Wild-type (W303-1A) and *ScNatA*Δ yeast cells transformed with empty pBEVY plasmid, wild-type SpNatA, or SpNatA ΔN-K6E were grown to early log-phase in SD-Ura medium. Ten-fold serial dilutions were spotted onto YPD and SD-Ura agar plates and incubated for 2 days at 30 or 38 °C. wt; wild-type

The NatA complex is conserved from yeast to human, as shown by complementation of growth phenotypes and partial rescue of the N-terminal acetylome [35]. With this in mind, we developed a simple functional assay for NatA mutations that is based on the temperature-sensitive growth phenotype of budding yeast lacking NatA [35, 36, 47, 48]. To functionally assess the SpNaa15 ΔN K6E mutant, we transformed a *ard1*Δ*/nat1*Δ double deletion strain (*ScNatA*Δ) (Fig. 3a) with a bidirectional expression vector encoding either SpNatA wild-type or SpNatA ΔN K6E (schematic of the constructs is shown in Fig. 2c). SpNatA expression in the *ScNatA*Δ deletion strain was confirmed by immunoblot analysis using anti-HA and anti-V5 to detect HA-SpNaa15 and SpNaa10-V5, respectively (Fig. 3b). As expected the SpNaa15 ΔN K6E variant had a lower molecular weight than full-length SpNaa15 (HA-SpNaa15, 746 amino acids, 85.5 kDa). We also observed a non-specific band around 80 kDa, which is caused by the secondary antibody used.

We next performed a yeast growth assay where serial dilutions of wild-type (W303-1A) + empty vector, *ScNatA*Δ + empty vector, *ScNatA*Δ + SpNatA, or *ScNatA*Δ + SpNatA ΔN K6E strains was spotted onto YPD and selective SD-Ura media. In agreement with previous studies, we observed reduced growth of the NatA knockout strain at elevated temperatures (Fig. 3c). Moreover, overexpression of the SpNatA complex rescued the growth defect of *ScNatA*Δ mutant cells. Consequently, SpNatA can functionally replace ScNatA, suggesting that the NatA complex is structurally very similar in the two yeast species. This observation was made on both YPD and SD-Ura growth media. Thus, *ScNatA*Δ mutant cells are able to retain the SpNatA plasmid even in the absence of selection due to the high selective pressure on cell viability. Overexpression of the SpNatA ΔN K6E mutant, however, failed to rescue the temperature-sensitive growth phenotype of *ScNatA*Δ. Although the SpNatA ΔN K6E variant was enzymatically active in vitro [44], this finding indicates that ribosome binding is essential for normal NatA function in vivo. Favorable electrostatic interactions appear to be a common feature for the interaction between molecular chaperones involved in co-translational folding and the ribosome [43, 44]. The NatA complex associates with the ribosome in a salt-sensitive manner, indicating an association mediated by ionic interactions [40, 44, 49]. Intriguingly, both the positively charged N-terminal region and the positively charged internal helix in Naa15 are situated on the same side of NatA and facing the ribosomal exit tunnel [44]. Thus, modifications within these two regions may interfere with NatA’s ability to interact with the ribosome and perform co-translational N-terminal acetylation by affecting the orientation and the electrostatic potential. The binding to Naa10 and NAT-activity were not affected in vitro [44]. Nevertheless, overexpression of SpNatA ΔN K6E seems to confer *ScNatA*Δ cells with slightly better growth at 38 °C compared to empty vector, suggesting that this mutant may have a small residual capacity to acetylate selected substrates controlling this phenotype. It may either mean that the mutant has retained a weak ability to associate with ribosomes, or has a residual capacity to perform post-translational N-terminal acetylation.

In conclusion, this yeast model complements the findings from the Magin study and supports the notion that EPR1 and EPR2 of the ribosome binding subunit Naa15 plays important structural role for correct and efficient NatA activity during protein synthesis. Mutation in these electropositive regions could have deleterious effects and, as suggested by Magin et al. could be used for therapeutic targeting of NatA activity by modifying ribosome binding.

### Limitations

- We acknowledge that this study only provide preliminary insights into the interaction between NatA and the ribosome and that it does not exclude the possibility that other regions on the NatA surface or binding factors are important for ribosome binding. For example, the NatA complex also associates with Naa50 [40] and in multicellular eukaryotes the chaperone-like protein HYPK (Huntingtin-interacting protein K) [50].
- We focused on SpNatA ΔN K6E since Magin et al. [44] showed that this mutant displayed the most severe ribosomal pulldown effect.
- Incorporation of a V5-tag C-terminal of SpNaa10 and a HA-tag N-terminal of SpNaa15 may affect SpNatA complex formation, ribosome binding, and/or enzymatic activity, but wild-type and mutant SpNaa15 were assayed and compared with identical tags.
- We have not performed any SpNatA-ribosome pulldown experiments using yeast lysates.

## Abbreviations

EPR: electropositive region
Naa: Nα-terminal acetyltransferase
NAT: N-terminal acetyltransferase
Sc: *Saccharomyces cerevisiae*
Sp: *Schizosaccharomyces pombe*
TPR: tetratricopeptide repeats
